# Virome comparisons in wild-diseased and healthy captive giant pandas

**DOI:** 10.1186/s40168-017-0308-0

**Published:** 2017-08-07

**Authors:** Wen Zhang, Shixing Yang, Tongling Shan, Rong Hou, Zhijian Liu, Wang Li, Lianghua Guo, Yan Wang, Peng Chen, Xiaochun Wang, Feifei Feng, Hua Wang, Chao Chen, Quan Shen, Chenglin Zhou, Xiuguo Hua, Li Cui, Xutao Deng, Zhihe Zhang, Dunwu Qi, Eric Delwart

**Affiliations:** 1 Department of Microbiology, School of Medicine, Jiangsu University, Zhenjiang, Jiangsu 212013 China; 2 Sichuan Key Laboratory of Conservation Biology for Endangered Wildlife, Chengdu Research Base of Giant Panda Breeding, Chengdu, Sichuan 610081 China; 3 Department of Swine Infectious Disease, Shanghai Veterinary Research Institute, Chinese Academy of Agricultural Sciences, Shanghai, 200241 China; 4 Department of Laboratory Medicine, Jiangsu Taizhou People’s Hospital, Taizhou, Jiangsu 225300 China; 5 School of Agriculture and Biology, Shanghai Jiaotong University, Shanghai, 200240 China; 6 Blood Systems Research Institute, Department of Laboratory Medicine, University of California San Francisco, San Francisco, CA 94118 USA; 7 Sichuan Key Laboratory of Conservation Biology for Endangered Wildlife, Chengdu Research Base of Giant Panda Breeding, Chengdu, Sichuan 610000 China

**Keywords:** Giant panda, Viral metagenomics, Virome, Papillomavirus, Picornavirus, Anellovirus, Gemycircularvirus, Putative circovirus

## Abstract

**Background:**

The giant panda (*Ailuropoda melanoleuca*) is a vulnerable mammal herbivore living wild in central China. Viral infections have become a potential threat to the health of these endangered animals, but limited information related to these infections is available.

**Methods:**

Using a viral metagenomic approach, we surveyed viruses in the feces, nasopharyngeal secretions, blood, and different tissues from a wild giant panda that died from an unknown disease, a healthy wild giant panda, and 46 healthy captive animals.

**Results:**

The previously uncharacterized complete or near complete genomes of four viruses from three genera in *Papillomaviridae* family, six viruses in a proposed new *Picornaviridae* genus (Aimelvirus), two unclassified viruses related to posaviruses in *Picornavirales* order, 19 anelloviruses in four different clades of *Anelloviridae* family, four putative circoviruses, and 15 viruses belonging to the recently described *Genomoviridae* family were sequenced. Reflecting the diet of giant pandas, numerous insect virus sequences related to the families *Iflaviridae*, *Dicistroviridae*, *Iridoviridae*, *Baculoviridae*, *Polydnaviridae*, and subfamily *Densovirinae* and plant viruses sequences related to the families *Tombusviridae*, *Partitiviridae*, *Secoviridae*, *Geminiviridae*, *Luteoviridae*, *Virgaviridae*, and *Rhabdoviridae*; genus *Umbravirus*, *Alphaflexiviridae*, and *Phycodnaviridae* were also detected in fecal samples. A small number of insect virus sequences were also detected in the nasopharyngeal secretions of healthy giant pandas and lung tissues from the dead wild giant panda. Although the viral families present in the sick giant panda were also detected in the healthy ones, a higher proportion of papillomaviruses, picornaviruses, and anelloviruses reads were detected in the diseased panda.

**Conclusion:**

This viral survey increases our understanding of eukaryotic viruses in giant pandas and provides a baseline for comparison to viruses detected in future infectious disease outbreaks. The similar viral families detected in sick and healthy giant pandas indicate that these viruses result in commensal infections in most immuno-competent animals.

**Electronic supplementary material:**

The online version of this article (doi:10.1186/s40168-017-0308-0) contains supplementary material, which is available to authorized users.

## Background

The giant panda (*Ailuropoda melanoleuca*) is the sole strictly herbivorous bear species [1] within *Ursidae* family [2], whose other members are carnivores or omnivores. The State Forestry Administration of China reported 1864 wild giant pandas in the fourth national survey, representing a 16.8% increase over the previous decade resulting in its re-categorization as vulnerable in the International Union for Conservation of Nature’s Red List of Threatened Species. Wild panda habitat and protected areas have significantly expanded in the last decade to 5.94 million hectares; however, anthropogenic habitat loss (e.g., deforestation) and poaching still threaten this vulnerable species [3]. The current global population of captive giant pandas is 425, approaching the population development goal of 500.

A low reproductive success and infectious diseases have hampered the development of captive and wild populations of the giant panda. Changes in the population or habitat of giant pandas may place animals at increased risk of infectious disease and hinder conservation efforts. The future of the endangered giant pandas depends in part on the development of protective measures against infectious diseases, especially viral infection. Although multiple studies of the bacterial content of giant panda feces have been reported [4–8], virologic study of giant panda has been limited to the identification and pathogenicity of single viruses including canine distemper virus, canine parvovirus, and influenza H1N1 [9–11].

Viral metagenomics has enabled the discovery of viral pathogens, viruses of unknown pathogenicity, and viruses of unknown cellular origins [12, 13]. In this method, the concentration of host-derived and bacterial background nucleic acids in biological samples is reduced by filtration and nuclease digestion while viral nucleic acids are protected within capsids. Random amplification of DNA and RNA followed by deep sequencing generates metagenomics sequence information from which viral sequences are identified by translated sequence protein similarity searches to all known eukaryotic viral sequences.

## Methods

### Sample collection and preparation

In April 2015, a wild giant panda was found dying in Tangjiahe Nature Reserve in Guangyuan, Sichuan Province. The sick giant panda died after transportation to the nearest Dujiangyan giant panda first-aid center. This wild giant panda adult showed clinical signs including extreme emaciation (half of normal adult weight), extreme dehydration, anemia, mouth mucous membrane canker, and ascaris nematode worms. Serologic test indicated it was weakly positive for canine parvovirus antibody. In order to elucidate whether a viral infection was related to this animal’s condition, feces, blood and nasopharyngeal secretion were collected prior to its death and heart, liver, spleen, lung, and kidney tissues collected after its death. In May 2015, another fresh fecal sample from an apparently healthy wild giant panda was collected from Tangjiahe Nature Reserve. Twenty-five fecal samples, 11 whole blood samples, and 10 nasopharyngeal secretion swabs were also collected from 46 clinically normal captive giant pandas in Chengdu Research Base of Giant Panda Breeding in Sichuan Province, China, from January 2014 to May 2015. All samples were collected by disposable materials and shipped on dry-ice. Fecal samples were re-suspended in ten volumes of phosphate-buffered saline (PBS) and vigorously vortexed for 5 min. Fecal supernatants were then collected after centrifugation (10 min, 15,000×*g*). The tips of respiratory swabs were immersed into 1 mL PBS and vigorously vortexed for 5 min and incubated for 30 min in 4 °C. The supernatants were then collected after centrifugation (10 min, 15,000×*g*). Tissue samples (~25 mg) were homogenized, frozen, and thawed three times on dry-ice, and the supernatants were then collected after centrifugation (10 min, 15,000×*g*). The whole-blood samples were centrifuged (10 min, 15,000×*g*) for the collection of plasma. Sample collection and all experiments in the present study were performed with the ethical approval given by the Ethics Committee of Jiangsu University and the reference number is No. UJS2014017.

### Viral metagenomic analysis

Five hundred microliters of each supernatant was filtered through a 0.45-μm filter (Millipore) to remove eukaryotic and bacterial cell-sized particles. The filtrates enriched in viral particles were treated with DNase and RNase to digest unprotected nucleic acid at 37 °C for 60 min [14–16]. Remaining total nucleic acid was then isolated using QiaAmp Mini Viral RNA kit (Qiagen) according to the manufacturer’s protocol. Forty-one libraries were then constructed using Nextera XT DNA Sample Preparation Kit (Illumina) and sequenced using the MiSeq Illumina platform with 250 bases paired ends with dual barcoding for each pool. The information of each library is shown, where most of the libraries were constructed based on individual samples, except for 8 out of the 11 fecal libraries of the normal captive giant pandas consisting of 3 or 4 pooled fecal samples (Table 1). For bioinformatics analysis, paired-end reads of 250 bp generated by MiSeq were debarcoded using vendor software from Illumina. An in-house analysis pipeline running on a 32-node Linux cluster was used to process the data. Reads were considered duplicates if bases 5 to 55 were identical and only one random copy of duplicates was kept. Clonal reads were removed and low sequencing quality tails were trimmed using Phred quality score ten as the threshold. The unique read number of each library was shown in Table 1. Adaptors were trimmed using the default parameters of VecScreen which is NCBI BLASTn with specialized parameters designed for adapter removal. The cleaned reads were de novo assembled within each barcode using the ENSEMBLE assembler [17]. Contigs and singlets reads are then matched against a customized viral proteome database using BLASTx with an *E* value cutoff of <10^−5^, where the virus BLASTx database was compiled using NCBI virus reference proteome (ftp://ftp.ncbi.nih.gov/refseq/release/viral/) to which was added viral protein sequences from NCBI nr fasta file (based on annotation taxonomy in Virus Kingdom). Candidate viral hits are then compared to an in-house non-virus non-redundant (NVNR) protein database to remove false-positive viral hits, where the NVNR database was compiled using non-viral protein sequences extracted from NCBI nr fasta file (based on annotation taxonomy excluding Virus Kingdom). Contigs without significant BLASTx similarity to viral proteome database are searched against viral protein families in vFam database [18] using HMMER3 [19–21] to detect remote viral protein similarities. A web-based graphical user interface was developed to present users with the virus hits, along with taxonomy information and processing meta-information. The genome coverage of the target viruses were analyzed by Geneious (Biomatters).

**Table 1:** Library information of samples of giant pandas included in the present study

Library ID	Sample type	No. of Sample	Healthy status	Habitation	Total no. of raw reads	No. of unique reads
WB1	Blood	1	Sick	Wild	361,812	29,382
CB2	Blood	1	Normal	Captive	434,190	31,095
CB3	Blood	1	Normal	Captive	18,834	2852
CB4	Blood	1	Normal	Captive	2,527,742	275,698
CB5	Blood	1	Normal	Captive	157,152	9274
CB6	Blood	1	Normal	Captive	428,670	29,353
CB7	Blood	1	Normal	Captive	11,236	1887
CB8	Blood	1	Normal	Captive	126,664	34,738
CB9	Blood	1	Normal	Captive	2,199,190	156,333
CB10	Blood	1	Normal	Captive	491,388	38,996
CB11	Blood	1	Normal	Captive	227,730	26,883
CB12	Blood	1	Normal	Captive	176,224	21,045
15FSW	Feces	1	Sick	Wild	194,826	70,181
16FNW	Feces	1	Normal	Wild	528,634	164,066
17FNC	Feces	3	Normal	Captive	977,118	534,051
18FNC	Feces	3	Normal	Captive	1,137,802	779,197
19FNC	Feces	3	Normal	Captive	976,318	541,294
20FNC	Feces	3	Normal	Captive	1,608,664	1,137,893
21FNC	Feces	3	Normal	Captive	1,222,508	933,678
22FNC	Feces	3	Normal	Captive	1,290,834	906,394
23FNC	Feces	3	Normal	Captive	2,906,028	1,526,096
24FNC	Feces	4	Normal	Captive	588,344	467,682
25FNC	Feces	1	Normal	Captive	542,450	323,462
26FNC	Feces	1	Normal	Captive	213,834	73,624
27FNC	Feces	1	Normal	Captive	326,788	201,519
NSW1	Oral swab	1	Sick	Wild	292,802	120,260
NSC2	Oral swab	1	Normal	Captive	192,224	64,507
NSC3	Oral swab	1	Normal	Captive	321,150	99,124
NSC4	Oral swab	1	Normal	Captive	898,688	434,150
NSC5	Oral swab	1	Normal	Captive	208,236	53,208
NSC6	Oral swab	1	Normal	Captive	613,348	337,452
NSC7	Oral swab	1	Normal	Captive	1,266,946	194,751
NSC8	Oral swab	1	Normal	Captive	235,500	62,890
NSC9	Oral swab	1	Normal	Captive	362,618	103,994
NSC10	Oral swab	1	Normal	Captive	319,506	88,356
NSC11	Oral swab	1	Normal	Captive	176,696	77,722
THE	Heart	1	Sick	Wild	574,430	84,312
TLI	Liver	1	Sick	Wild	546,204	109,832
TSP	Spleen	1	Sick	Wild	447,030	35,613
TLU	Lung	1	Sick	Wild	274,480	48,401
TKI	Kidney	1	Sick	Wild	353,176	55,164

### PCR confirmation and genome sequencing

PCR confirmation was performed for the papillomavirus, gemycircularvirus, adenovirus, and insect viruses in the nasopharyngeal secretion samples; picornavirus, gyrovirus, and circoviruses in the fecal samples; gemycircularvirus in the blood samples; and anellovirus and the insect virus (Sacbrood virus) in the tissues. Inverse PCR were used to generate the complete genome of the novel anelloviruses in the blood samples and the gyrovirus in the fecal sample. PCR to bridge sequence gaps were used to acquire the complete genome of the picornaviruses in fecal samples and papillomavirus in nasopharyngeal secretion samples. Sequences and characteristics of the primers used in the present study are shown in Additional file 1: Table S1. Sanger method was used for sequencing of the PCR products.

### Quality control in the nucleic acid manipulation

Standard precautions were used for all procedures to prevent the cross-sample contamination and nucleic acid degradation. Mainly, aerosol filter pipet tips were used to reduce the possibility of sample cross contamination, and all the materials (including microcentrifuge tubes, pipet tips) which directly contacted with nucleic acid samples were RNase and DNase free. The nucleic acid samples were dissolved in DEPC-treated water.

### Phylogenetic analysis

Phylogenetic analyses were performed based on the predicted amino acid or nucleotide sequences in the present study, their closest viral relatives based on the BLASTx search in GenBank, and representative members of related viral species or genera. Sequence alignment was performed using CLUSTAL W with the default settings. Phylogenetic trees with 500 bootstrap resamples of the alignment data sets were generated using the Maximum-likelihood (ML) method in MEGA6.0. Bootstrap values (based on 500 replicates) for each node were given.

### Other sequence analysis

Putative ORFs in the viral genome were predicted by Geneious 8.1 software or NCBI ORF finder. Putative exon and intron were predicted by Netgenes2 at http://www.cbs.dtu.dk/services/NetGene2/. The conserved domains of the Pansaviruses (a novel virus in the order *Picornavirales*) from the feces of giant pandas were determined using the NCBI conserved domain search in combination with the Pfam conserved domain search [22, 23]. Mapping raw data to the reference virus genome was performed using the low sensitivity/fastest parameter in Geneious software version 8.1, where the mapped reads show >90% similarity to the reference genome.

### Nucleotide sequence accession numbers

The novel viral genomes described in detail here were deposited in GenBank under the following accession numbers MF327529–MF327579. The raw sequence reads from the viral metagenomic libraries were deposited in the Short Read Archive of GenBank database under the accession number SRX2882233.

## Results

### Overview of Virome

**Fig. 1:**
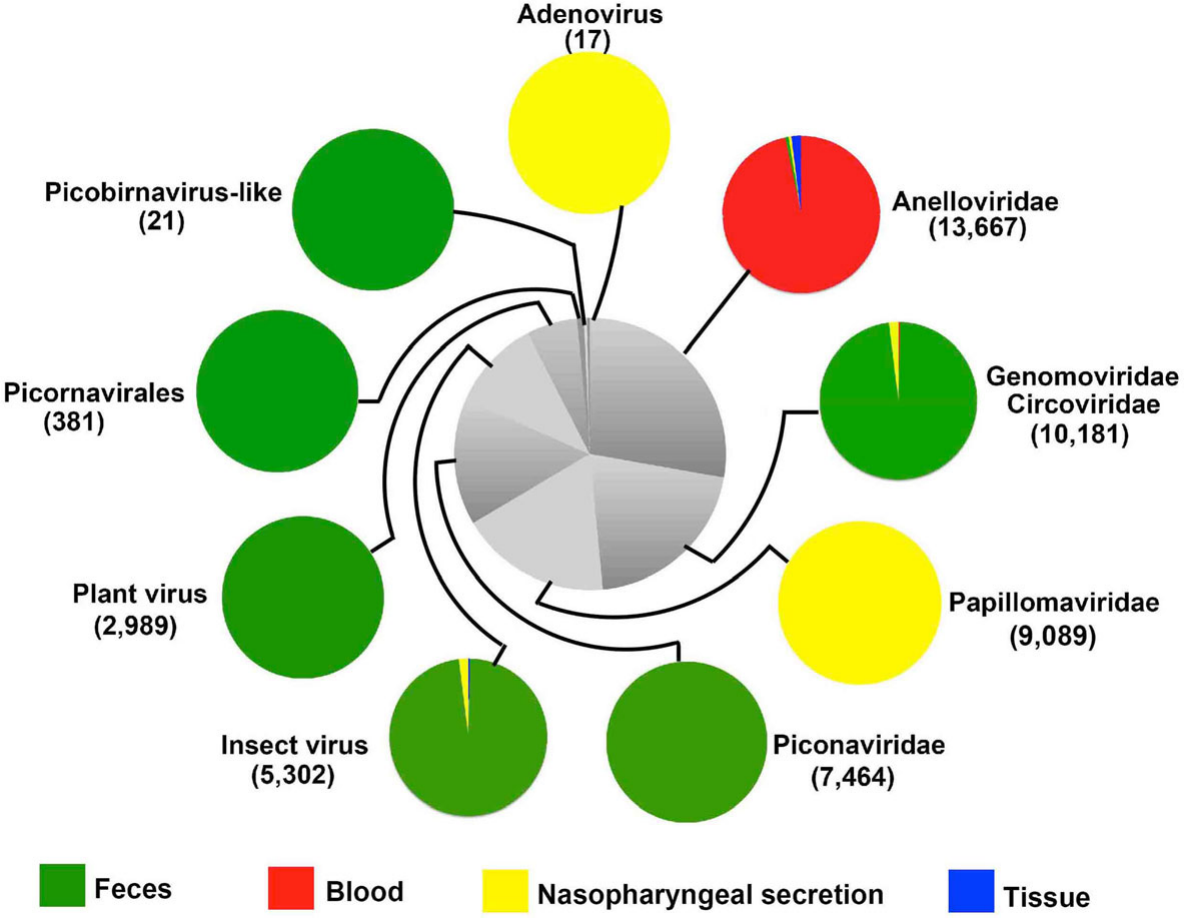
The composition and sample distribution of eukaryotic viruses detected in giant pandas. The pie chart in the center showed the approximate percentages of the nine virus groups detected in all types of sample. The nine circumjacent smaller pie charts indicated the approximate percentage of virus sequence from different type samples. Sample types were showed in *different colors*

Forty-one nucleic acid libraries from samples of giant pandas were generated and sequenced. The raw sequence reads numbers of each library generated by the Illumina MiSeq are shown in Table 1. Raw sequence reads were binned by barcodes and quality-filtered, leaving high-quality sequence reads which were de novo assembled within each barcode. The resulting sequence contigs and singlets were compared with the viral reference database and the GenBank non-redundant protein database using a BLASTx search with an E value cutoff of <10^−5^. Translated sequences similar to those of known or suspected eukaryotic viral proteins are summarized in Table 2. In the fecal samples we detected eukaryotic viral sequences related to *Picornaviridae*, *Genomoviridae*, *Circoviridae*, *Anelloviridae*, *Picobirnaviridae*, other unclassified virus in *picornavirales*, and numerous insect and plant viruses. The eukaryotic viral sequences detected in blood samples included anellovirus genomes present in each of the 12 plasma derived libraries together with a small number of viral sequences showing similarity to gemycircularviruses in the newly described *Genomoviridae* family. In the nasopharyngeal secretion samples, we detected eukaryotic viral sequences related to *Papillomaviridae*, *Anelloviridae*, *Genomoviridae*, and *Adenoviridae* families and a low number of insect viral sequences. In four of the five tissues from the diseased animal, sequences related to *Anelloviridae* family were detected. A small number of insect viruses were also detected in the lung tissue. The overall composition of eukaryotic viruses detected in this study is represented in Fig. 1.

**Table 2:** Eukaryotic viral sequences identified in the feces, blood, nasopharyngeal secretions, and tissues samples

Library ID	No. of unique reads	Reads no. of different viral species/families								
		Aenlloviridae	Adenoviridae	Genomo/Circoviridae	Picrnaviridae	Papillomaviridae	Posavirus-like	Picobirnavirus-like	Plant virus	Insect virus
WB1	29,382	3463	0	0	0	0	0	0	0	0
CB2	31,095	428	0	0	0	0	0	0	0	0
CB3	2852	61	0	0	0	0	0	0	0	0
CB4	275,698	530	0	6	0	0	0	0	0	0
CB5	9274	293	0	0	0	0	0	0	0	0
CB6	29,353	466	0	0	0	0	0	0	0	0
CB7	1887	26	0	0	0	0	0	0	0	0
CB8	34,738	10	0	0	0	0	0	0	0	0
CB9	156,333	4860	0	5	0	0	0	0	0	0
CB10	38,996	383	0	0	0	0	0	0	0	0
CB11	26,883	2411	0	0	0	0	0	0	0	0
CB12	21,045	584	0	0	0	0	0	0	0	0
15FSW	70,181	3	0	22	3309	0	74	0	15	70
16FNW	164,066	20	0	1258	1611	0	286	0	49	125
17FNC	534,051	0	0	234	63	0	21	0	31	155
18FNC	779,197	0	0	726	314	0	0	0	867	246
19FNC	541,294	0	0	926	177	0	0	0	602	86
20FNC	1,137,893	0	0	771	170	0	0	0	259	1234
21FNC	933,678	0	0	114	8	0	0	0	16	233
22FNC	906,394	8	0	2370	79	0	0	0	221	198
23FNC	1,526,096	3	0	793	1382	0	0	24	2619	358
24FNC	467,682	0	0	474	26	0	0	0	84	113
25FNC	323,462	0	0	18	230	0	0	0	13	14
26FNC	73,624	0	0	223	74	0	0	0	304	122
27FNC	201,519	0	0	2196	21	0	0	0	14	35
NSW1	120,260	14	2	0	0	8507	0	0	0	0
NSC2	64,507	0	0	0	0	0	0	0	0	0
NSC3	99,124	0	0	11	0	0	0	0	0	0
NSC4	434,150	0	0	8	0	321	0	0	0	114
NSC5	53,208	0	0	0	0	0	0	0	0	0
NSC6	337,452	0	0	57	0	0	0	0	0	48
NSC7	194,751	0	0	0	0	0	0	0	0	0
NSC8	62,890	0	0	0	0	0	0	0	0	0
NSC9	103,994	0	0	15	0	0	0	0	0	29
NSC10	88,356	0	0	0	0	0	0	0	0	0
NSC11	77,722	4	15	0	0	261	0	0	0	0
THE	84,312	5	0	0	0	0	0	0	0	0
TLI	109,832	13	0	0	0	0	0	0	0	0
TSP	35,613	56	0	0	0	0	0	0	0	0
TLU	48,401	19	0	0	0	0	0	0	0	17
TKI	55,164	0	0	0	0	0	0	0	0	0

### Comparison of the eukaryotic viruses in different giant pandas

**Fig. 2:**
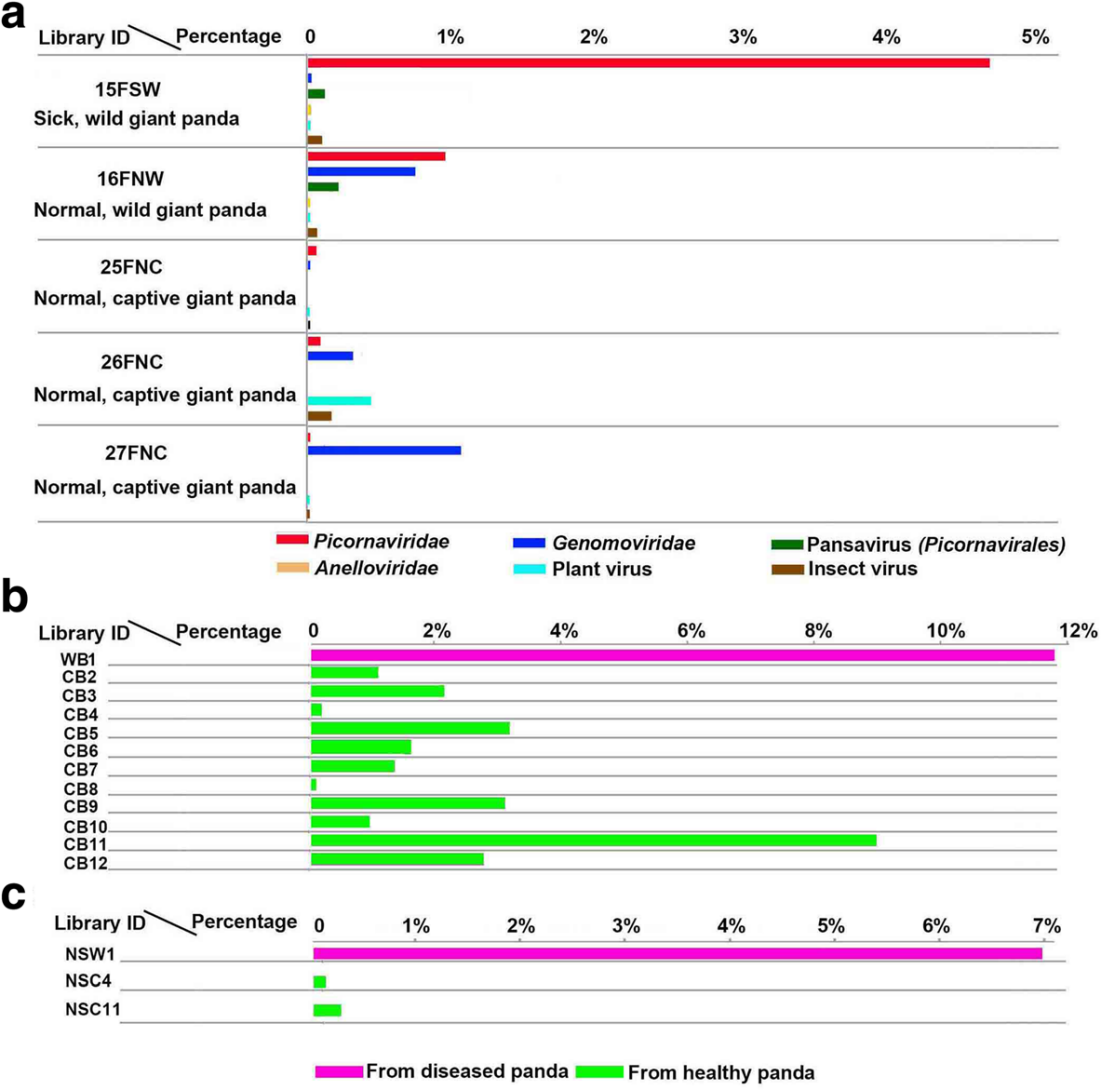
Virome comparisons of different samples of giant pandas based on BLASTx to the GenBank non-redundant database (E value of <10–5). **a** Percentage of virus-like sequence reads with similarity to eukaryotic viruses in the five libraries constructing based on five individual fecal samples. Viruses belonging to different groups were marked with *different colors*. **b** Percentage of anellovirus-related sequences in blood samples of different individual animals, where the bar related to the wild giant panda was marked with *green* and those related to the captive ones were marked with *purple*. **c** Percentage of papillomavirus-related sequences in the positive nasopharyngeal secretion swabs, where the bar related to the diseased giant panda was marked with *purple* and those related to captive ones were marked with *green*

The composition of eukaryotic viruses of feces from the sick wild giant panda, the healthy wild giant panda, and three individually sequenced healthy captive giant pandas (25FNC, 26FNC, and 27FNC) is shown (Fig. 2a). All five samples were positive for picornaviruses, with the feces from the sick wild animal showing the highest percentage (4.7%) relative to those of the other giant pandas (0.1–0.98%) (Fig. 2a). Anellovirus was detected in all 12 plasma samples, with the sample from the sick wild giant panda showing the highest percentage of anellovirus sequences (11.8%) while the percentage of anellovirus sequences in the other 11 samples ranged from 0.03 to 8.96% (Fig. 2b). The nasopharyngeal secretion sample of the sick wild giant panda contained abundant papillomavirus sequence reads whose sequence percentage (7.07%) was higher than those of the only two of ten nasopharyngeal secretions from healthy captive animals that were positive for papillomavirus (with sequence percentages of 0.07 and 0.3%, respectively) (Fig. 2c).

### Papillomaviruses in nasopharyngeal secretions of giant panda

Papillomaviruses (PVs) are a highly diverse family of double-stranded circular DNA genomes approximately 8 kb in size known to infect a wide variety of mammals, as well as birds and reptiles. Some PV types cause benign or malignant epithelial tumors of the skin and mucous membranes in their hosts, while others are commonly detected on healthy human skin, as well as on that of different animals. Papillomaviruses are classified into genera based on the sequence of the highly conserved ORF L1 [24]. While the overwhelming majority of PVs only infect epithelium and are highly host specific [24, 25], the bovine papillomaviruses (BPVs) of the *Deltapapillomavirus* genus have the ability to infect both epithelial and mesenchymal cells and to infect multiple species [26]. Recently, more complete genomes of PVs were reported in non-human species, including one from the oral mucosa of a polar bear [27] the closest extant relative of giant panda.

**Fig. 3:**
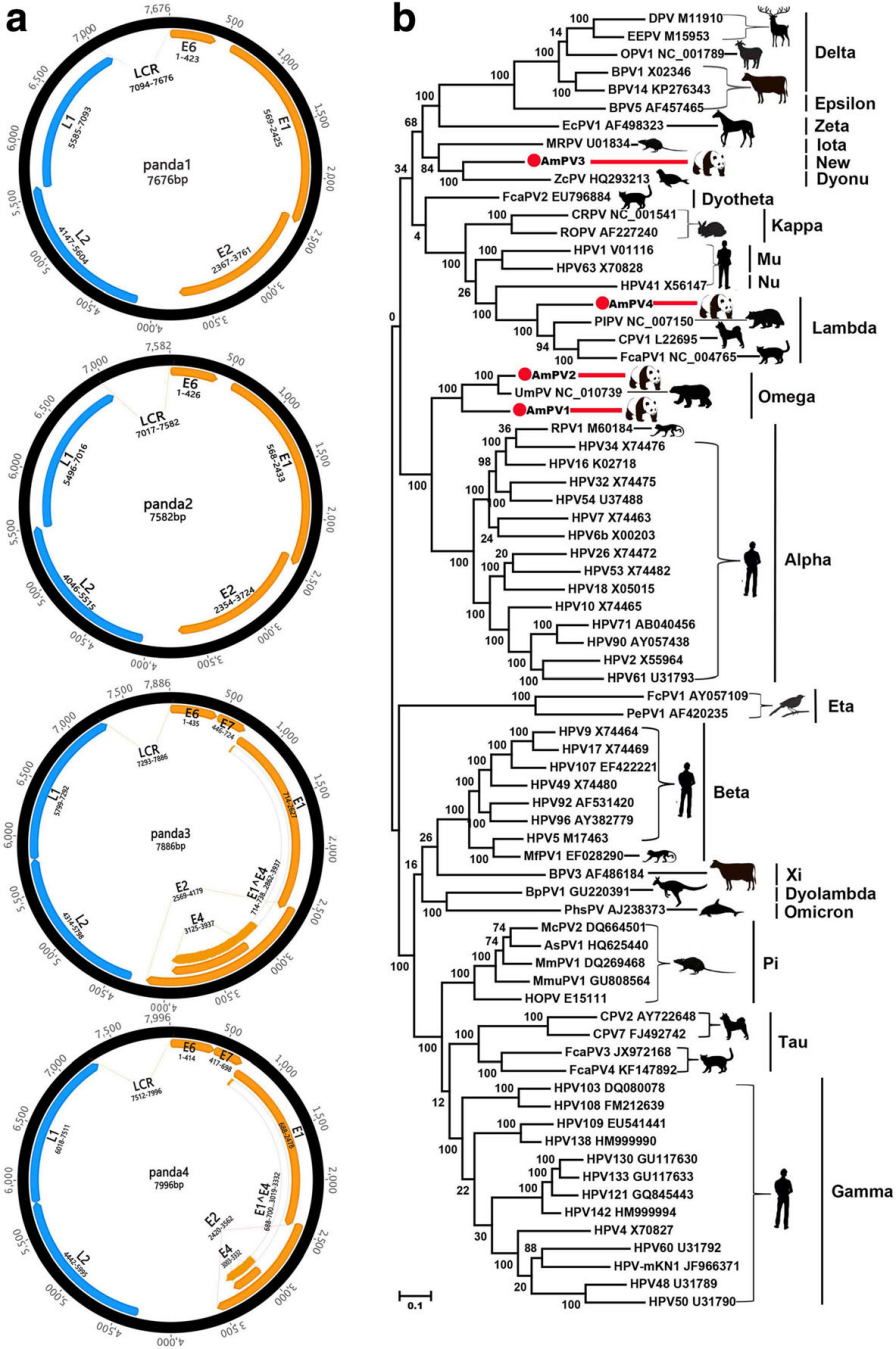
Genomic organization and phylogenetic analysis of the Papillomaviruses identified in the giant pandas. **a** Genomic organization of the AmPVs identified in the nasopharyngeal secretions of giant pandas. **b** Phylogenetic analysis was performed based on the amino acid sequence of L1 protein. The sequence alignments included the four AmPVs identified here, the best BLASTp matches in GenBank based on the L1 proteins of the AmPV1–4, and 66 representative species from each of the currently recognized genera. Silhouettes of the hosts included in the phylogenetic analysis were showed on *branches*. Papillomaviruses identified in this study was labeled with *red dots*

Here, we characterized four complete PV genomes from the nasopharyngeal secretions of giant pandas (*A. melanoleuca*), hereafter referred to as AmPV1, AmPV2, AmPV3, and AmPV4. The genomes of the four AmPVs are described in Additional file 2: Table S2. AmPV1 and AmPV2 were both found in the nasopharyngeal secretion of the diseased wild giant panda and shared 62.5% nucleotide sequence similarity over the complete genome. AmPV3 and AmPV4 were detected in the nasopharyngeal secretions from the two healthy captive giant pandas, respectively, and showed <55% sequence similarities to each other and AmPV1 and AmPV2 over the complete genome based on pairwise comparison. Genome sizes of the four AmPVs were 7676, 7582, 7886, and 7996 bp, with GC contents of 43.5, 45.6, 58.6, and 38.5%, respectively. Five distinct ORFs on the same coding strand were identified in all four PV genomes, including the early genes E6, E1, and E2 and the late genes L2 and L1 (Fig. 3a). Analysis of the deduced amino acid sequences revealed that many of the classic PV-specific elements were present in the four AmPVs (Additional file 2: Table S2).

To determine the divergence in sequence between the AmPVs found here and other PVs, amino acid sequence alignment of the complete L1 protein was performed, and an ML phylogenetic tree was generated. The sequence alignment included the four AmPVs identified here, the best BLASTp matches in GenBank based on the L1 proteins, and 66 representative species from each of the currently recognized genera [28]. Phylogenetic analysis indicated that AmPV1 and AmPV2 fell within a separate clade also including UmPV identified in the oral mucosa of a polar bear [27] (Fig. 3b). AmPV1 had the L1 identities of 73.6 and 73.1% with those of AmPV2 and UmPV1, respectively, while AmPV2 had identity of 91.1% with the L1 of UmPV1. AmPV3 clustered with ZcPV (from California sea lion), sharing 62.3% L1 protein sequence identity. AmPV4 clustered with PIPV, CPV1, and FcaPV1 from different carnivores, respectively, sharing L1 protein sequence identities of 71.4, 68.3, and 68.2%.

According to the International Committee on Taxonomy of Viruses (ICTV), papillomaviruses are classified using the conserved ORF L1 nucleotide sequence with PVs within the same genus expected to have greater than approximately 60% nucleotide identity and PVs within the same species greater than 70% identity. AmPV1 and AmPV2 both have the closest relative of UmPV1 (GenBank no. NC_010739) in GenBank and cluster together within *Omegapapillomavirus* (Fig. 3b). Based on the ORF L1 nucleotide sequence, AmPV2 has similarity of 80.3% to UmPV1, while AmPV1 has similarities of 69.6 and 68.5% to AmPV2 and UmPV1, respectively. Therefore, AmPV1 and AmPV2 together with UmPV1 belong to *Omegapapillomavirus*, where AmPV1 is a proposed new PV species while AmPV2 and UmPV1 belong to two different types within the same species. BLASTN search in GenBank with ORF L1 nucleotide sequence indicated that AmPV3 has the best match of ZcPV (GenBank no. HQ293213) and shares 58.3% similarity with it, suggesting that AmPV3 belongs to a proposed new genus. Based on the ORF L1 nucleotide sequence, AmPV4 has the best BLASTN searching match with PlPV (GenBank no. NC_007150), sharing identity of 68.1%, which suggests AmPV4 belongs to a new species within *Lambdapapillomavirus* genus.

### New picornavirus genus in feces of giant panda

**Fig. 4:**
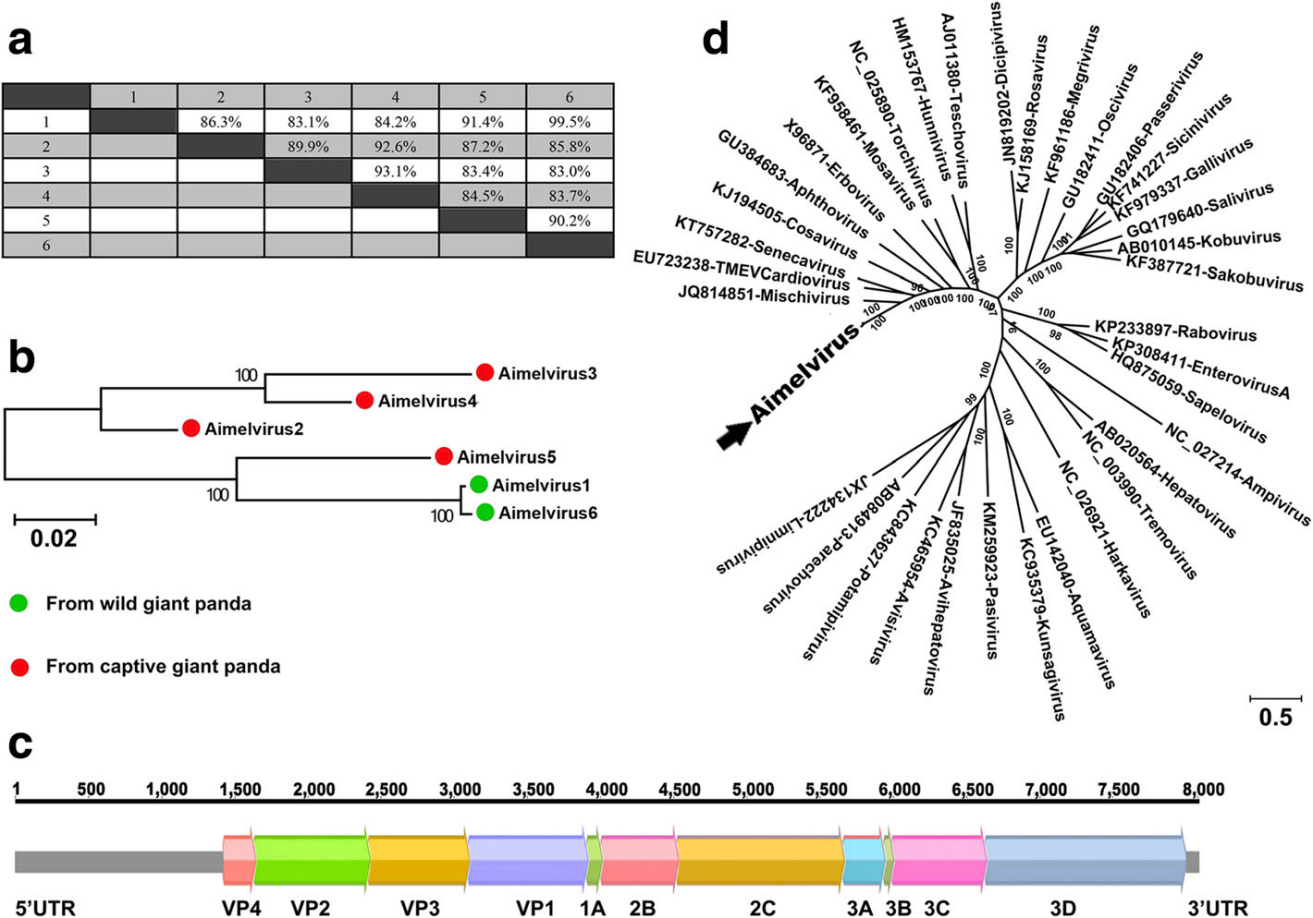
Sequence comparison, genomic organization, and phylogenetic analysis of the novel picornavirus identified in the giant pandas. **a** Sequence similarity among the six novel picornaviruses (Aimelvirus1–6) were compared. **b** Un-rooted phylogenetic tree showed the genetic relationship of Aimelvirus1–6. **c** Genomic organization was showed in *different colors*. **d** Phylogenetic analysis based on the complete amino acid sequence of P1 proteins of Aimeilvirus 1–6, and 35 representative strains of all the 35 genera in *Picornaviridae*

Picornaviruses are small, non-enveloped, positive-sense, single-stranded RNA viruses with a genome size of 7.1 to 8.9 kb, encoding a single polyprotein. The family *Picornaviridae* belongs to the order *Picornavirales* and currently consists of at least 54 species grouped into 31 genera. Additionally, there are four proposed new genera (each containing a single proposed species) and 24 proposed new species within existing genera (www.picornaviridae.com). Here, we characterized a novel picornavirus in fecal samples, which was provisionally named Aimelvirus (*Ailuropoda melanoleuca* virus). All libraries of fecal samples were positive for Aimelvirus. Six different complete genomes of Aimelvirus could be generated from six different fecal libraries, including two strains (Aimelvirus 1 and 6) from wild giant pandas and four strains (Aimelvirus 2–5) from captive animals, with genome size of 8003–8100 bp. The six Aimelviruses shared 83–99.5% sequence similarity (Fig. 4a) but showed no significant similarity with any viruses in GenBank over the full genomes based on BLASTn search. To investigate the relationship of the six Aimelviruses, a phylogenetic tree was established over the full-genome sequences (Fig. 4b), which showed that the six Aimelviruses were grouped into two separate clusters, one including three strains from captive giant pandas and the other one including one strain from captive giant pandas and two from the two wild giant pandas. In the lower cluster, although the two strains from the wild animals shared 99.5% nucleotide sequence similarity, they only showed 91.4 and 90.2% similarities to the strain from the captive animals.

The genomic organization of the six Aimelviruses is typical of picornaviruses, with a single large ORF encoding the complete polyproteins of 2191–2218 amino acids (aa) in length. The polyprotein comprised the capsid proteins VP4, VP2, VP3, and VP1 and non-structural proteins 2A to 2C and 3A to 3D (Fig. 4c). According to the ICTV (http://www.picornastudygroup.com/definitions/genus_definition.htm), the members of a picornavirus genus should share >40, >40, and >50% amino acid similarity in their P1, P2, and P3 regions, respectively. The P1 regions of the six Aimelviruses shared 37.9–39.0% amino acid similarities with their closest relative, Saffold virus; the P2 and P3 regions of them shared 38.0–38.6% and 46.7–47.0% amino acid similarities, respectively, with their closest relative, the African bat icavirus. Therefore, according to the genetic distance-based criteria, the Aimelviruses from the giant pandas can be considered a novel genus in the family of *Picornaviridae*. Phylogenetic analyses were performed on the P1 and P3 regions of Aimelviruses here and 35 representative strains of all 35 genera in *Picornaviridae*, indicating that Aimelvirus was not significantly linked to any recognized or proposed genera (Fig. 3d and Additional file 3: Figure S1).

### A novel virus in *Picornavirales* order in feces of giant panda

Members of the order *Picornavirales* are non-enveloped, single-stranded positive-sense RNA viruses found in a wide range of protozoa, plants, and animals. The order is currently composed of five recognized families: *Picornaviridae*, *Secoviridae*, *Iflaviridae*, *Marnaviridae*, and *Dicistroviridae* in addition to a large number of proposed, unassigned members. Conserved features found in all *Picornavirales* include non-structural proteins consisting of helicase, chymotrypsin-like protease, and RNA-dependent RNA polymerase (RdRp) domains. Genomic organization is variable with a single polyprotein expressed in all orders except *Dicistroviridae* where structural and non-structural protein genes are expressed from separate transcripts [29]. Recently, some highly divergent members of the *Picornavirales* order have been discovered, including porcine-stool-associated RNA viruses (posaviruses) in the feces of pigs [30–32], fish-stool-associated RNA virus (fisavirus) in the intestinal content of a healthy carp [33], and human-stool-associated virus (husavirus) [34].

**Fig. 5:**
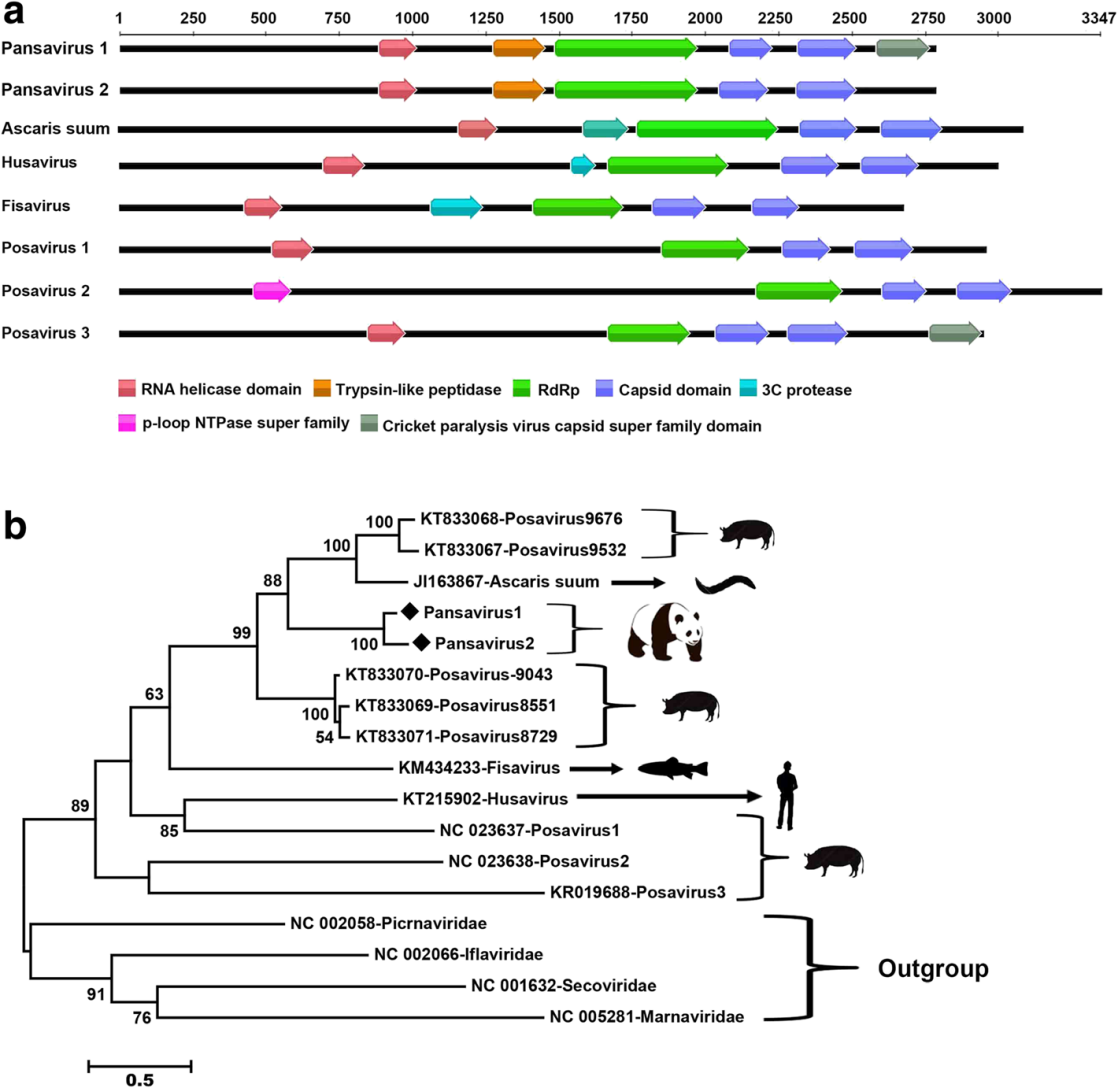
Genomic organization and phylogenetic analysis of the novel picornavirales identified in the giant pandas. **a** Positions of conserved domains within the polyproteins of pansaviruses and their related viruses were shown in *different colors*. **b** Phylogenetic analysis was performed based on the amino acid sequence of RDRP protein. The sequence alignments included the two pansaviruses identified here and related strains in GenBank. Silhouettes of the hosts included in the phylogenetic analysis were showed on *branches*. Pansaviruses identified in this study were labeled with *diamond*

In the present study, two large contigs, 8703 and 8612 bp, both encoding a 2778 aa polyprotein which showed sequence similarity to the polyprotein of posaviruses, were detected in the fecal samples from both wild giant pandas. We provisionally named the viruses Pansavirus 1 and Pansavirus 2 (panda-stool-associated RNA viruses 1 and 2). Based on the complete polyprotein, the two pansaviruses shared 82.1% amino acid identity to each other. In the polyprotein of Pansavirus 1, five conserved domains could be identified by an NCBI combined with a Pfam conserved domain search, while in the polyprotein of Pansavirus 2, only four conserved domains could be identified (Fig. 5a). The RdRp and capsid proteins of pansavirus both shared the highest amino acid identity of about 48% with the Posavirus strain 9676. The phylogenetic relationship was then determined by aligning the putative-encoded RDRP protein with representative members of the *Picornavirales*. Pansaviruses clustered together with other five posaviruses and a transcript from the nematode Ascaris suum, yet is clearly distant from posaviruses 1–3, fisavirus, and husavirus (Fig. 5b).

### Anelloviruses in the blood samples of giant pandas

Anelloviruses are non-enveloped, with circular single-stranded DNA genome of 2.1–3.9 kb in length depending on the isolate analyzed. Anelloviruses are subgrouped into 12 genera including 3 infecting humans, namely Alphatorquevirus or Torque teno virus (TTV), Betatorquevirus or Torque teno mini virus (TTMV), and Gammatorquevirus or Torque teno midi virus (TTMDV) [35]. Anelloviruses are widely prevalent in humans and animals and except for a possibly pathogenic porcine anellovirus [36] most appear to be commensal infections whose concentration increases with decreased immune function [37–39].

Here, abundant sequence reads were detected with translated amino acid similarity to viruses in the family *Anelloviridae*. We named the viruses Giant panda Anellovirus (GpAV). GpAV sequence reads were detected in all 12 blood samples, 4 of the 13 feces libraries, 2 of the 11 nasopharyngeal secretions, and 4 of the 5 tissues. From the 12 blood libraries abundant of anellovirus sequence reads, 19 complete genomes were got by de novo assembly combining inverse PCR and direct Sanger sequencing of the amplicons. The genome sizes of the GpAVs were ranged from 1964 to 2663 bp, whose circular genomes are depicted in Additional file 4: Figure S2. These genomes showed similar genomic organization in their ORF1, ORF2, and ORF3; although, ORF3 could not be detected in one genome and the locations of other theoretical ORFs of unknown function differed. ORF1 sequences of the 19 GpAVs ranged from 441 aa to 685 aa long, with typical arginine-rich regions at their N-termini. Sequence analysis indicated that the 19 GpAVs shared sequence identity of their ORF1 proteins ranging from 23 to 56%.

**Fig. 6:**
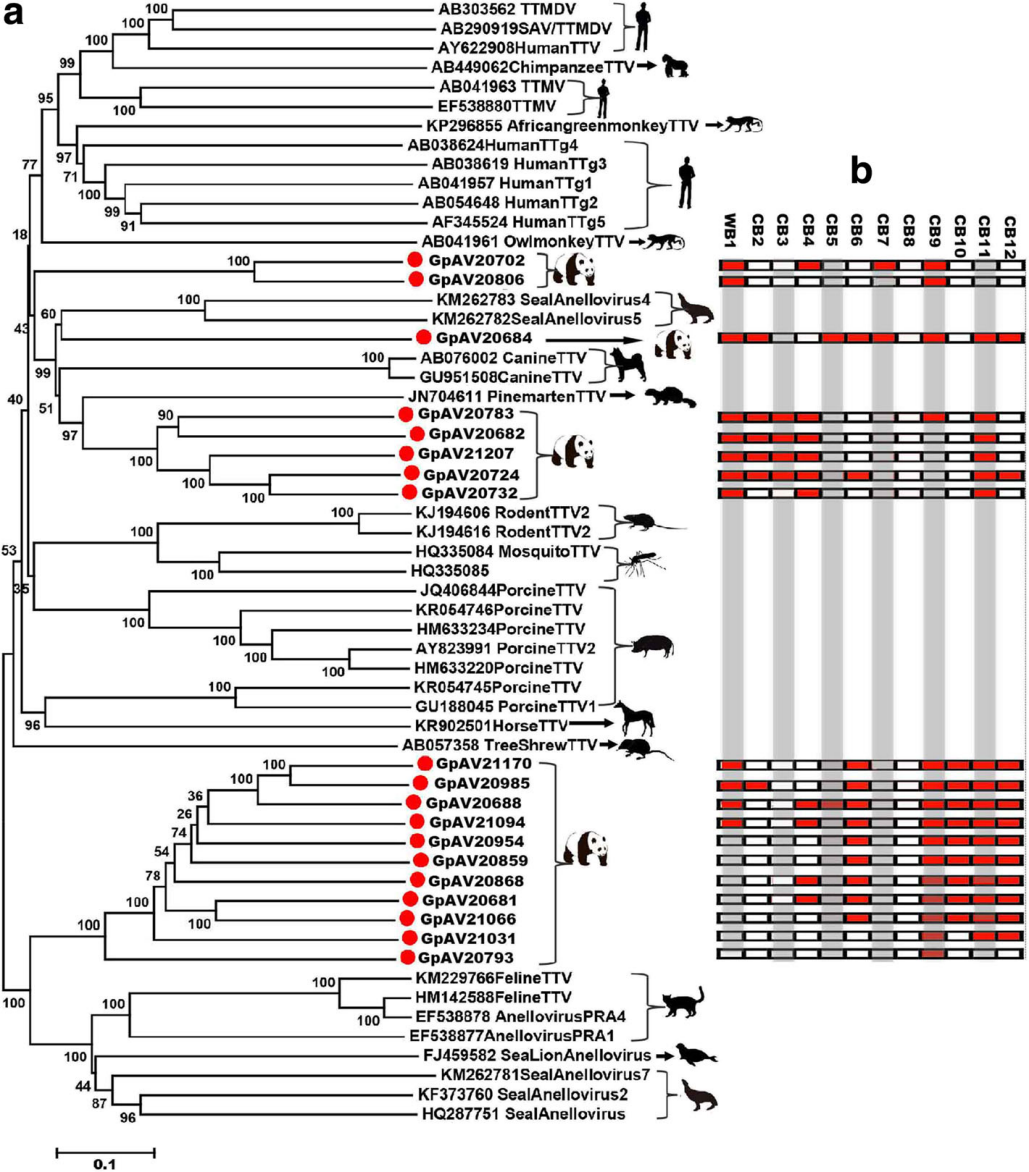
Phylogenetic analysis and co-infection of the novel anelloviruses identified in the blood samples of giant pandas. **a** Phylogenetic analysis was performed based on the amino acid sequence of ORF1 protein. The sequence alignments included the 19 anelloviruses identified here, their best BLASTp matches in GenBank based on the ORF1 proteins, and the representative anellovirus strains from GenBank. Silhouettes of the hosts included in the phylogenetic analysis were showed on *branches*. Anelloviruses identified in this study were labeled with *red dots*. **b** The co-infection of anellovirus in blood giant pandas. The 12 columns and 19 rows were set corresponding to 12 blood samples and 19 anelloviruses, respectively, with complete genomes. The *small box with red color* stands for positive, and the *white box* stands for negative

Phylogenetic analyses were performed based on the ORF1 amino acid sequences of the 19 GpAVs, their best BLASTp matches in GenBank and the representative members of related viruses. Results indicated that the 19 GpAVs could be located in four different clades, including one, two, five, and 11 GpAV strains, respectively (Fig. 6a). In order to detect co-infections in blood samples, the raw sequencing data of the 12 libraries from blood samples were aligned to each of the 19 complete genomes of GpAV, where the 19 genomes were used as reference genomes, the libraries with >3 different reads matching to the reference genome were considered as positive for the reference strain. High levels of co-infection of GpAV were detected in blood samples of giant pandas, with individual animals carrying highly distinct anellovirus variants (Fig. 6b). Co-infections were detected in 11 of the 12 blood samples. Library CB9 from a healthy captive animal had the highest level of co-infection, carrying 15 different GpAV strains. The library WB1 from the diseased wild giant panda was co-infected with 12 different GpAV strains (Fig. 6b).

### Circoviridae-like and Genomoviridae in multiple samples of Giant pandas

*Circoviridae*, including recognized genera Circovirus and Cyclovirus are non-enveloped, single-stranded circular DNA (≈2 kb) viruses. *Genomoviridae*, currently consisting of a single genus Gemycircularvirus, is a new family of eukaryote-infecting single-stranded (ss) circular ssDNA viruses [40]. Circoviruses are thought to exhibit host species specificity and have been detected in various species, including birds and mammals, and associated with a variety of diseases particularly in pigs, including respiratory and enteric disease, dermatitis, and reproductive problems [41–44]. Gemycircularviruses were found in a wide range of organism and environments [44, 45]. Only one cellular host, a fungus [46], has been definitely identified for a single gemycircularvirus (SsHADV-1). Other gemycircularvirus genomes have been detected in fungi infected plants [47], sewage [48], the bodies of insects [45, 49], as well as mammalian feces and tissues [50, 51]. More recently, gemycircularviruses were described from human blood from blood donors [15], an HIV-positive patient [52], and human cerebral spinal fluid and feces [53]. Both circoviruses and gemycircularviruses have an ambisense genome organization with two major inversely arranged open reading frames encoding the rolling circle replication initiator protein gene (Rep) and a capsid protein gene (Cap). A conserved stem-loop structure, required for viral replication, is located between the 5′ ends of the two main ORFs.

**Fig. 7:**
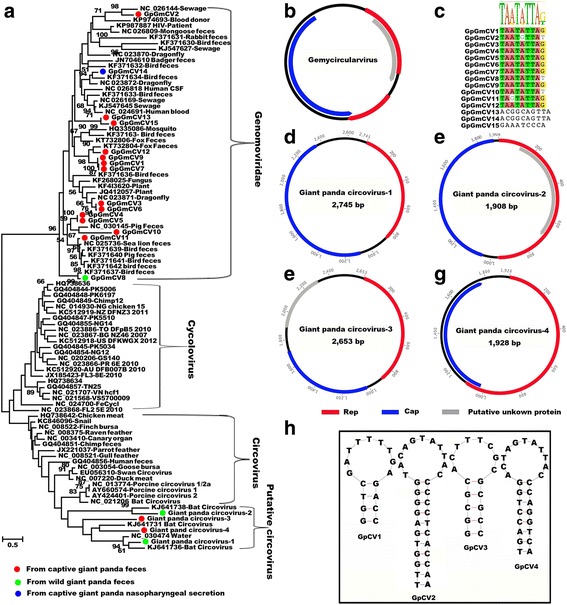
Phylogenetic analysis and genomic organization of the novel gemycircularviruses and putative circoviruses identified in the giant pandas. **a** Phylogenetic analysis was performed based on the amino acid sequence of Rep protein. The sequence alignments included 15 gemycircularviruses and 4 putative circoviruses identified here, their best BLASTp matches in GenBank based on the Rep proteins, and the representative strains of gemycircularvirus and circovirus. Hosts or sources of these viruses included in the phylogenetic analysis were showed on *branches*. Viruses identified in this study were labeled with *colored dots*. **b** The consensus genomic organization of the gemycircularviruses identified in giant pandas. **c** The nonamer in stem-loop structure of gemycircularviruses identified in this study. **d**–**g** The genomic organizations of the four GpCVs. **h** The stem-loop structures of the four GpCVs identified in giant pandas

Here, gemycircularvirus sequence reads were detected in feces, nasopharyngeal secretions and blood samples, while circovirus-related sequences were only detected in the fecal samples. Fifteen novel gemycircularvirus genomes, named giant panda gemycircularvirus (GpGmCV), and four genomes related to circoviruses, named giant panda circovirus-like viruses (GpCV), were assembled from these libraries. Among the 15 genomes of gemycircularvirus, 14 were from fecal samples and one (GpGmCV14) from nasopharyngeal secretion. Among the 14 GpGmCV genomes from fecal samples, 13 were from captive giant pandas and one (GpGmCV8) from the wild healthy giant panda (Fig. 7a). The four GpCV genomes were all from fecal samples, two of which were from the healthy wild giant panda while the other two from two different captive animals (Fig. 7a). The 15 genomes of GpGmCVs, with genome size of 2063 to 2240 bp long, have similar genome organizations, including a spliced Rep protein ORF, a Cap ORF in the opposite orientation, and a putative unknown protein on the same orientation of Rep (Fig. 7b). The stem-loop structure was found between the 5′ ends of the two main ORFs of the genomes of all the 15 GpGmCVs, 12 of which contained a conserved nonamer of “TARTRTTK”, while the other three have distinct base composition (Fig. 7c). The four genomes of GpCV have genome size of 1908 to 2745 bp long, whose ORF organizations are different though they all contain the two mains proteins of Rep and Cap (Fig. 7d–g). GpCV1 and GpCV4 have the Rep protein and the Cap protein in the same orientation but GpCV2 and GpCV3 contain the two major proteins in the opposite orientation that is the classical genome feature of circovirus. The four GpCVs have different stem-loop structures within the non-coding region between the ORFs of Rep and Cap, only two of which include the typical circovirus nonamer origin of replication (T/cAGTATTAC) (Fig. 7h).

In order to phylogenetically classify the GpGeCVs and GpCVs, their Rep proteins were aligned to those of circoviruses, cycloviruses, and gemycircularviruses. The phylogenetic analysis included the GpGeCVs and GpCVs identified in the present study and their best BLASTp matches based on Rep in GenBank, and representative strains of circoviruses, cycloviruses, and gemycircularviruses. Results indicated that the 15 GpGmCVs were located on 8 genetically different separate branches (Fig. 7a), sharing 45–87% identities with their closest relatives. Although the four GpCVs were genetically very divergent based on the amino acid sequence of Rep, they nonetheless clustered together with four other putative circoviruses including three circovirus-like viruses from bat and one circovirus-like virus from a Chinese lake sample, sharing 35–62% amino acid sequence identity (Fig. 7a).

### RNA virus related to both picobirnavirus and partitivirus

Picobirnavirus (PBV), the only genus in the new *Picobirnaviridae* viral family, has a bi-segmented dsRNA genome. The large RNA segment encodes the capsid protein while the small segment encodes the viral RdRp. PBV was originally found in the intestines of rat and has since been found in numerous mammals, birds, and reptiles [54]. Members of family *Partitiviridae* are characterized by having genomes comprised of two linear, monocistronic dsRNA segments (1.4 to 2.4 kbp in length) [55], where the smaller codes for the CP and the larger codes for the virion-associated RNA polymerase. Most partitiviruses examined to date are associated with latent infections of their fungal, plant, and protozoan hosts [55].

**Fig. 8:**
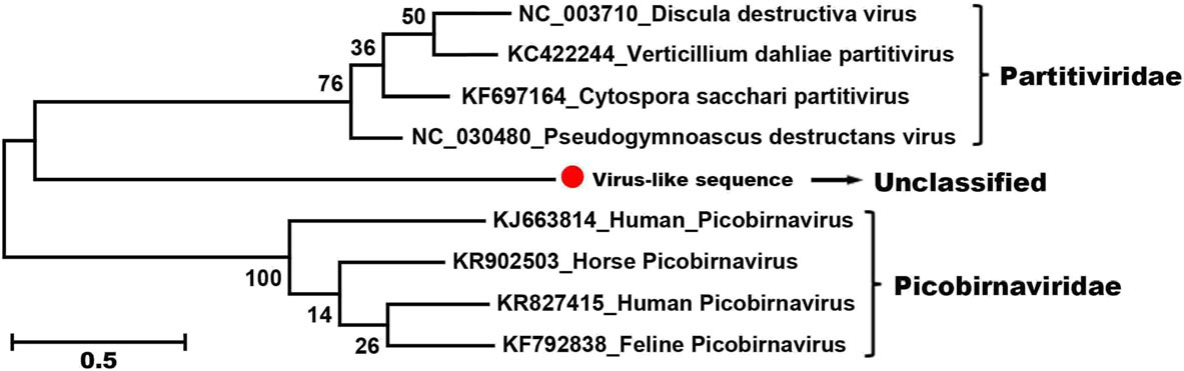
Phylogenetic analysis of the partitivirus-like sequence identified in the giant pandas. Phylogenetic analysis was performed based on the amino acid sequence of RDRP protein. The sequence alignments included the partitivirus-like sequences identified in the giant pandas, four representative partitiviruses, and four representative picobirnaviruses. The partitivirus-like sequence identified in this study was labeled with a *red dot*

In the present study, one fecal library (ID: 23FNC) contained 24 RdRp sequence reads that assembled into a full-length RdRp gene whose amino acid sequence showed approximately 30% identity to the viruses from both *Paritiviridae* and *Picobirnaviridae*. Alignment of full-length RdRp allowed the construction of a phylogenetic tree using four representative best BLASTx matches of partitivirus and picobirnavirus, in GenBank. This RdRp sequence phylogenetically falls between partitivirus and picobirnavirus (Fig. 8). Identification of a segment encoding a capsid gene failed, which may due to a high level of sequence divergent. Sequence mapping using this RdRp sequence as a reference to the raw data of this library showed that it is also present in other two fecal libraries. Whether this RdRp sequence belongs to the *Partitiviridae* or *Picobirnaviridae* families or is a new viral family is currently unknown.

### Other mammalian viruses

Other viral genomes with high-sequence similarity to known viruses were also detected. Gyroviruses have been proposed as members of *Anelloviridae* as they share common genome characteristics [56]. Fifty-nine gyrovirus reads were detected in a fecal library (ID: FCN21) generated from three fecal samples from captive animals which could be assembled into three contigs. The complete genome was determined by inverse PCR, which contained 2375 bp and shared the highest sequence identity of 97.8% with an avian gyrovirus (AGV2 GenBank: HM590588) detected in chicken serum [57] and human feces in China and 96% identity with a related genome found on human skin [58]. In the nasopharyngeal secretion sample from the sick wild giant panda, 15 sequence reads also showed similarity (70–90%) to adenovirus proteins which could be assembled into five contigs ranged 272 to 472 bp matching five different gene regions of Mastadenovirus.

### Insect and plant viruses

A large number of reads related to insect viruses were detected in all the fecal libraries. A smaller number of insect virus reads were detected in three nasopharyngeal secretion samples and the lung tissue (Table 2). Detection of these viral sequences in fecal samples may be due to insect consumption or the presence of intestinal parasites including nematodes. The presence of insect viral sequences in nasopharyngeal secretion samples and the lung tissue may be due to inhalation of insect-contaminated material or parasite infection in the respiratory tract. For instance, the largest contig (604 bp) assembled from the 29 reads in one nasopharyngeal secretion sample (ID: NSC9) showed amino acid sequence identity of 88% (*E* value = 4e^−101^) with the structural protein of a *Drosophila immigrans* Nora virus (NC_024488). The insect genome in the lung tissue belongs to a divergent member of the *Iflaviridae* family, with the largest contig (600 bp) sharing an amino acid sequence identity of 30% (*E* value = 7e^−21^) with the structural protein (VP1) of Sacbrood virus (KX254334). Overall, the insect RNA virus-like reads were more abundant than those of insect DNA viruses, making up >70% of insect viral sequences. Most of the RNA sequences were related to the members of the *Iflaviridae* and *Dicistroviridae*. Insect DNA viruses were mainly from the viral families *Iridoviridae*, *Baculoviridae*, and *Polydnaviridae* and subfamily *Densovirinae*. The insect virus sequences shared amino acid sequence identity of 25–90% with annotated insect viral proteins.

Large numbers of plant virus sequences were detected in each fecal library but none in respiratory swabs, plasma, or tissues (Table 2). RNA viruses were predominant with >80% of plant viral reads, the majority being related to single-stranded RNA viruses in the family *Tombusviridae*, followed by the families *Partitiviridae*, *Secoviridae*, *Geminiviridae*, *Luteoviridae*, *Virgaviridae*, and *Rhabdoviridae*, genus *Umbravirus*, and family *Alphaflexiviridae*. Most of the plant DNA viral reads were related to members of the *Phycodnaviridae* family.

## Discussion

Animal and human viral discovery has long been focused on pathogenic infections and viruses that could be readily grown in cell cultures and cause visible cytopathic effects. Viral metagenomics is a recent approach to analyzing mixtures of viral nucleic acids enriched directly from a variety of sources without a prerequisite for amplification in tissue culture. Viral metagenomics has recently been used in numerous animal virus discoveries [14, 30, 59–62], providing information on the composition of animal viromes, helping to provide candidates to identify the etiology of infectious disease in animals and identify zoonotic and emerging viruses. We describe here the eukaryotic viral communities in the feces, blood, nasopharyngeal secretion, and five tissues of giant pandas. The animals analyzed here consisted of an extremely emaciated wild giant panda who died of an unknown cause, one healthy wild giant panda, and 46 healthy captive giant pandas. The highest percentage reads of a new picornavirus in feces and of anelloviruses in plasma (19 different variants) were detected in the sick animal relative to all healthy giant pandas. The nasopharyngeal swab of the diseased animal also generated the largest percentage of papillomavirus reads relative to the only two papillomavirus positive healthy animals. A low level of adenovirus reads was also found in nasopharyngeal secretion. The nasopharyngeal swab of the diseased animal contained three mammalian viruses (two types of papillomavirus, anellovirus, and adenovirus), higher than those of the healthy captive giant pandas with an average of 0.9 distinct mammalian viruses per animal (with co-infection ranging from 0 to 3). The diseased animal was co-infected with at least 12 different anelloviruses, a greater number than the average of seven distinct anellovirus per animal (range 0–15).

The picornavirus and papillomavirus stood out as two viruses whose sequence reads percentages were much higher than those of the healthy giant pandas. Increases in viral sequence reads concentration might reflect weakened immune status of the diseased animal as is known to occur with anelloviruses in humans [37–39]. Although serologic test indicated that this sick wild giant panda was weakly positive for canine parvovirus antibodies, none of its samples showed the presence of canine parvovirus sequences.

Papillomaviruses are believed that have co-evolved with their vertebrate host species, a hypothesis supported by the fact that PVs of closely related host species are generally closely related themselves [63, 64]. In the present study, four PVs (AmPV1–4) belonging to three different genera were detected in the nasopharyngeal secretion of the sick wild and two captive giant pandas. AmPV1 and AmPV2 were detected in the nasopharyngeal secretion sample of the diseased giant panda and phylogenetically clustered with the UmPV both in the *Omegapapillomavirus* genus (Fig. 3b) from the oral mucosa of a polar bear with papillomas on the tongue [27]. The two different AmPVs generated sequences accounting for a large 7.07% of the unique sequence reads (Fig. 2c), which may have contributed to the mouth mucous membrane canker of the dying wild giant panda [65]. A *Lambdapapillomavirus* was recently associated with oral papillomas in southern sea otters which may have interfered with their feeding capacity [66]. AmPV3 and AmPV4 were found in two nasopharyngeal secretion samples from two captive giant pandas, respectively. AmPV4 showed the highest sequence similarity to PIPV detected in the a cutaneous lesion biopsy of a raccoon [67], both of which phylogenetically grouped into the genus *Lambdapapillomavirus* also containing CPV1 from dogs and FcaPV1 from cats (Fig. 3b). AmPV3, although clustering with PVs from rat and sea lions (Fig. 3b), may represent a new genus in *Papillomaviridae* family based on ICTV genetic distance criteria. The AmPV1, AmPV2, and AmPV4 clustered with their closest relatives, UmPV and PIPV, whose hosts, polar bears and raccoons are close relative of giant panda. With the increasing number of PVs discovery from animals, we anticipate that the relative of AmPV3 will be detected in hosts closely related to giant pandas.

The genome of a tentative new *picornaviridae* genus, provisionally labeled Aimelvirus, was characterized. The greatest sequence similarity values were seen with members of the genera *Mischivirus*, *Cardiovirus*, and *Senecavirus*. We generated six complete or near complete genomes of Aimelviruses. These genomes could be classified into two potential species, each with three strains. Two strains from two wild animals clustered together showing high sequence identity (99.5%) and 91.4% to the other strain in the same clade from a captive animal (Fig. 4a). The three strains in the other group were all from the captive giant pandas and showed more sequence divergence, sharing sequence similarity of 89.9–93.1% among themselves. All fecal libraries, including eight libraries constructed from small sample pools and five from individual sample, were positive for Aimelvirus (Table 2), suggesting that giant pandas are frequent natural hosts of this picornavirus. None of the blood samples were positive for Aimelvirus.

The order *Picornavirales* includes viruses replicating in all the major divisions of eukaryotes, including protozoans, plants, insects, and mammals [68]. In the current study, two novel viral RNA genomes with distant sequence relationships to *Picornavirales* were characterized and provisionally named pansaviruses. Phylogenetic analysis based on the amino acid sequence of RdRp indicated that these genomes clustered together with those of posaviruses from pig feces and the transcriptome of an Ascaris suum transcriptome (Fig. 5b) [32]. Since an *Ascaris suum* transcriptome sequence was also grouped into this cluster, pansaviruses may not actually infect the intestinal cells of the giant panda but a nematode species in the gut of these animals, a potential source supported by the clinical record that the diseased giant panda contained ascarids in its intestine. The possible origin of posaviruses from pigs from aquatic algae has also been postulated based on their similarity to members of the algae infecting *Marnaviridae* and detection in water from wells on pig farms [32]. Only one fecal sample from the 25 captive giant pandas was positive for pansavirus, possibly reflecting the different environment in a breeding center relative to the wild.

In recent years, many genomes of *rep*-containing circular DNA viruses in the *Circoviridae* and *Genomoviridae* have been characterized in mammals, birds, insects, fungi, and environmental samples bringing to light a high level of genetic diversity among these viruses [40, 44, 56]. In this work, we reported the discovery of 19 novel circular ssDNA genomic sequences from giant panda, 15 belonging to gemycircularvirus in *Genomoviridae* and 4 possibly belonging to circovirus in *Circoviridae*. Gemycircularviruses have been reported in feces of a wide range of organism and different environments [40, 45]; however, the host species of these viruses are not known except for one replicating in a fungi. In this study, based on the Rep protein sequence, some gemycircularviruses from giant panda samples showed high sequence identity to the viruses detected in insects or feces from other animals (Fig. 7a), e.g., GpGmCV3 and GpGmCV6 sharing identity of 81% with the gemycircularvirus from dragonfly (NC_023871) [45] and GpGmCV8 sharing 87% sequence identity with gemycircularvirus from bird feces (KF371637) [69], which suggested that the detection of gemycircularvirus genomes in giant panda may be from consumed fungi or insects. As for the four putative circoviruses, two (GpCV3 and GpCV4) were from captive giant pandas and the other two (GpCV1 and GpCV2), from the healthy wild giant panda.

Anelloviruses from humans and animals show a very high level of genetic diversity [70–72]. Co-infections with multiple anellovirus variants simultaneously present in the same individual and animals have been described [73–76]. As induced, infectious, or inborn immunodeficiency is associated with increases in viral loads, anelloviruses are believed to be under immunological control [37, 38, 77, 78]. Anelloviruses have been reported in many tissues and bodily fluids, including respiratory fluids, blood, breast milk, cervical secretions, semen, urine, and feces [35]. In the present study, Anellovirus were detected in all studied sample types, with blood samples showing the highest sequence reads percentage. All the 12 blood samples were positive for anellovirus, 11 of which revealed co-infections. The diseased wild animal showed the highest percentage anellovirus sequence reads (11.8%) which may reflect this giant panda’s poor immunity in the late stage of its disease.

The virome is one part of the microbiome in mammals which is being continuously updated using viral metagenomic sequencing. The present study analyzed the viromes of a wild-diseased and multiple healthy giant pandas. The same viral families were detected in both the dying and the healthy animals; although, the fraction of papillomavirus, anellovirus, and picornavirus were greater in the affected animal. It is increasingly clear that the presence of viruses is not necessarily indicative of pathogenesis and instead may represent a degree of commensal interactions between virus and host [79]. It is difficult to know how stably associated the virome is with the host, as some viruses rely solely on a lytic life cycle and tend to produce short-lived acute infections. However, non-cytopathic viruses, which do not cause such profound cell death, can infect and persist for the lifespan of the host. In the present study, the Aimelvirus and Anellovirus were detected in all of the studied giant pandas including the healthy and sick animals. The replication of these viruses may increase if the host has low immunity [38], and further worsen the host’s condition. In this study, the virus titers of anelloviruses in the blood and Aimelvirus in the gut of the sick giant panda were distinctly higher than those from the normal giant pandas (Fig. 3), suggesting that the balance between the immune control and viral replication was compromised, leading to the higher viral loads in the already sick giant panda.

## Conclusion

Our study provides an overview of the virome of the giant pandas and significantly increases the diversity of viruses known to infect this vulnerable species. Comparison of the viruses in the healthy animals with those in future giant panda suffering sporadic or epidemic unexplained diseases of possible viral origins will help identify newly emerging, possibly pathogenic, viruses. Higher read number of papillomaviruses, anelloviruses, and picornaviruses in the emaciated, sick, animal may reflect reduced immune control of otherwise commensal infections.

## Additional files

Additional file 1: Table S1. Primers used for specific PCR confirmation and inverse PCR. (DOCX 24 kb)

Additional file 2: Table S2. Genome size, GC content, and classic elements of papillomavirus identified in giant pandas. Classic elements included positions of Zinc-binding domains of E6 and E7 ORFs, LxCxE motif of E7 ORF, ATP binding site of the ATP-dependent helicase (GPPDTGKS), E2-binding site motifs, TATA box, and polyadenylation site of URR. (DOCX 18 kb)

Additional file 3: Figure S1. Phylogenetic tree based on the P3 protein of Aimelvirus. Phylogenetic analysis was performed based on the complete amino acid sequence of P3 proteins of Aimeilvirus 1–6 and 35 representative strains of all the 35 genera in Picornaviridae. The Aimelvirus identified in this study was labeled with a black dot. (JPEG 368 kb)

Additional file 4: Figure S2. Genome structure of anelloviruses identified in the present study. (JPEG 1012 kb)

## Abbreviations

AmPV: *Ailuropoda melanoleuca* papillomavirus
BLAST: Basic Local Alignment Search Tool
GpAV: Giant panda anellovirus
GpCV: Giant panda circovirus
GpGmCV: Giant panda gemycircularvirus
