# Supplementary material for: Virome comparisons in wild-diseased and healthy captive giant pandas
Source: Microbiome. 2017 Aug 7;5:90. doi: 10.1186/s40168-017-0308-0 (PMC5545856; doi:10.1186/s40168-017-0308-0)
Supplement: Supplementary file 1 — Primers used for specific PCR confirmation and inverse PCR. (DOCX 24 kb) [file 40168_2017_308_MOESM1_ESM.docx]

**Table S1. Primers used for specific PCR confirmation and inverse PCR.**

| **Primer** | **Targeted virus** | **Application** | **Sequence (5´-3´)** | **Fragment size (bp)** | **Annealing temperature (oC)** | **No. of cycles** |
| --- | --- | --- | --- | --- | --- | --- |
| **AmpvS1 sense** | **AmPV1** | **PCR confirmation** | **TGGGCCTCCTAACACAGGTA** | **436** | **55** | **35** |
| **AmpvS1 antisense** |  |  | **GTCTAGCCTTGCCCACACTT** |  |  |  |
| **AmpvS2 sense** | **AmPV2** | **PCR confirmation** | **TTGGTGTGCATGAAAGCGTG** | **328** | **55** | **35** |
| **AmpvS2 antisense** |  |  | **GCTATGTGCTGCAACGCTAC** |  |  |  |
| **AmpvS3 sense** | **AmPV3** | **PCR confirmation** | **GTCGGGCCCAAAACAGAAAC** | **384** | **55** | **35** |
| **AmpvS3 antisense** |  |  | **TTGTCGCTTTTGAAGGTGCG** |  |  |  |
| **AmpvS4 sense** | **AmPV4** | **PCR confirmation** | **TGTGGAAGCAGAGTGTAGCG** | **364** | **55** | **35** |
| **AmpvS4 antisense** |  |  | **CCGAACACGCACATCTACCT** |  |  |  |
| **Ampv3In sense** | **AmPV3** | **Bridge PCR** | **AAGGCCGGGTTACTGAACTG** | **1938** | **52** | **38** |
| **Ampv3In antisense** |  |  | **GGAGTGGACAGTGGGATGTG** |  |  |  |
| **Ampv4In1 sense** | **AmPV4** | **Bridge PCR** | **TGCCTCGCATGCATAAGACT** | **1300** | **53** | **38** |
| **Ampv4In1 antisense** |  |  | **CAGCACTTCGAGCCCTAGG** |  |  |  |
| **Ampv4In2 sense** | **AmPV4** | **Bridge PCR** | **ATGCAAAGAAACCGCAAGCG** | **1299** | **55** | **38** |
| **Ampv4In2 antisense** |  |  | **CAGCTGCAAATCAGTGCCAA** |  |  |  |
| **Gemy14 sense** | **GpGmCV14** | **PCR confirmation** | **CGAACATTGTCCTGGGTCCA** | **317** | **55** | **35** |
| **Gemy14 antisense** |  |  | **CTCCGCTGTGTCGAACGATA** |  |  |  |
| **Adenos sense** | **Adenovirus** | **PCR confirmation** | **CTTCCATGGGCGTAAAATCG** | **239** | **52** | **35** |
| **Adenos antisense** |  |  | **GGTTTCCAGTCAAGCTCCGA** |  |  |  |
| **InsectVs1 sense** | **Drosophila immigrans Nora virus** | **PCR confirmation** | **CAACTCCACTCAAGAGCGGT** | **277** | **55** | **35** |
| **InsectVs1 antisense** |  |  | **GACTTTGCTTGGGACGGAGA** |  |  |  |
| **InsectVs2 sense** | **Sacbrood virus** | **PCR confirmation** | **ACACATCGCTACGCATGGAT** | **259** | **55** | **35** |
| **InsectVs2 antisense** |  |  | **CTGTCCCCAAGCTGTAACGT** |  |  |  |
| **Aimel1 sense** | **Aimelvirus1** | **PCR confirmation** | **ACAACAACTGCCTGGTGACA** | **228** | **53** | **35** |
| **Aimel1 antisense** |  |  | **CCTGTTTTGGGAGAGGGGAC** |  |  |  |
| **Aimel2 sense** | **Aimelvirus2** | **PCR confirmation** | **CACACAGTTCAGAGGGTCCC** | **437** | **53** | **35** |
| **Aimel2 antisense** |  |  | **CAACGGTTGTGGCATCACTG** |  |  |  |
| **Aimel3 sense** | **Aimelvirus3** | **PCR confirmation** | **ACAACTGCCCCTGACACAAA** | **294** | **53** | **35** |
| **Aimel3 antisense** |  |  | **GGCAGGTCCACAGAACATGA** |  |  |  |
| **Aimel4 sense** | **Aimelvirus4** | **PCR confirmation** | **TCTTCTGGTGTGGGAGACGA** | **316** | **53** | **35** |
| **Aimel4 antisense** |  |  | **TTACATCCCACAGTGGCCTG** |  |  |  |
| **Aimel5 sense** | **Aimelvirus5** | **PCR confirmation** | **CCATCCCTGTTACTGTGCGT** | **266** | **53** | **35** |
| **Aimel5 antisense** |  |  | **GTGACGGCCAGGTAAAGACA** |  |  |  |
| **Aimel6 sense** | **Aimelvirus6** | **PCR confirmation** | **GGTCCCCGAAGCCACATTTA** | **346** | **53** | **35** |
| **Aimel6 antisense** |  |  | **TCTCATGGGGGCCTGTCTAA** |  |  |  |
| **GyroS sense** | **Gyrovirus** | **PCR confirmation** | **GCTGGGGAAACAAGCCTTTG** | **395** | **52** | **35** |
| **GyroS antisense** |  |  | **TGACTCTGGACGCTATGGGA** |  |  |  |
| **GyroIv sense** | **Gyrovirus** | **Inverse PCR** | **G*C*CACCACTCACTCGGTGGTAC** | **2105** | **50** | **39** |
| **GyroIv antisense** |  |  | **G*C*CTTGTTTCCCCAGCTTTGC** | |  |  |
| **GpCVs1 senese** | **GpCV1** | **PCR confirmation** | **CTGGGGCAGAACTGGTACTG** | **276** | **55** | **35** |
| **GpCVs1 antisenese** |  |  | **TCAGGATACCACTGCTCTGGA** |  |  |  |
| **GpCVs2 senese** | **GpCV2** | **PCR confirmation** | **GTCTACATTGCAAGCGCTCG** | **317** | **55** | **35** |
| **GpCVs2 antisenese** |  |  | **GGATGTGGTCCAGGGACTTG** |  |  |  |
| **GpCVs3 senese** | **GpCV3** | **PCR confirmation** | **TGCCCAGTATTCGATGCAGG** | **309** | **55** | **35** |
| **GpCVs3 antisenese** |  |  | **ACTTCGAACTGACTGCCCAG** |  |  |  |
| **GpCVs4 senese** | **GpCV4** | **PCR confirmation** | **CAGCTTTGCAGGCACAACAA** | **372** | **55** | **35** |
| **GpCVs4 antisenese** |  |  | **CCTGGTAGTTGTCCCACCAC** |  |  |  |
| **Anel1Iv sense** | **GpAV20681** | **Inverse PCR** | **G*C*AAATGGGGACTCAGAAACGAA** | **1577** | **52** | **38** |
| **Anel1Iv antisense** |  |  | **G*C*ACCTGTTTGTTTGCTGGTGC** | |  |  |
| **Anel2Iv sense** | **GpAV20682** | **Inverse PCR** | **ATCCAAGTGGCAATGGGGAG** | **1468** | **52** | **38** |
| **Anel2Iv antisense** |  |  | **TATGTCAGGGTGAGGCCAGA** |  |  |  |
| **Anel3Iv sense** | **GpAV20684** | **Inverse PCR** | **CCGGAATTGCTTGCAGATCG** | **1389** | **52** | **38** |
| **Anel3Iv antisense** |  |  | **CCCTGGTTAGATGTGCTCCA** |  |  |  |
| **Anel4Iv sense** | **GpAV20688** | **Inverse PCR** | **G*C*ACCACCAATCCTGTCATCCG** | **1422** | **52** | **38** |
| **Anel4Iv antisense** |  |  | **G*C*CCAAGTGTCAGAGCAGGTGT** | |  |  |
| **Anel5Iv sense** | **GpAV20702** | **Inverse PCR** | **ACAAGTCGCTGAATGCCAGA** | **1258** | **52** | **38** |
| **Anel5Iv antisense** |  |  | **CGTCTAAAGGTCCTCGGACG** |  |  |  |
| **Anel6Iv sense** | **GpAV20724** | **Inverse PCR** | **GATGCTGCTCGATCAGACGA** | **1256** | **52** | **38** |
| **Anel6Iv antisense** |  |  | **CTGTGTTGTGGGTGCCATTG** |  |  |  |
| **Anel7Iv sense** | **GpAV20732** | **Inverse PCR** | **TACAGCGAAGAAAGCAGCGA** | **1278** | **54** | **35** |
| **Anel7Iv antisense** |  |  | **CGAAACCAAGGTCGAAAGCG** |  |  |  |
| **Anel8Iv sense** | **GpAV20783** | **Inverse PCR** | **ACAGCGAGCCATCAACAGAA** | **968** | **54** | **35** |
| **Anel8Iv antisense** |  |  | **AAAGCGTCGTCTCCTTACCG** |  |  |  |
| **Anel9Iv sense** | **GpAV20793** | **Inverse PCR** | **AGCTCACCCGCTTTATTCGA** | **838** | **54** | **35** |
| **Anel9Iv antisense** |  |  | **CCTTCTGCGGGTGATACTCC** |  |  |  |
| **Anel10Iv sense** | **GpAV20806** | **Inverse PCR** | **GCGCCTTACCGTTTGAAGAC** | **756** | **54** | **35** |
| **Anel10Iv antisense** |  |  | **CTGTAGTCTGCGGTGCCTG** |  |  |  |
| **Anel11Iv sense** | **GpAV20859** | **Inverse PCR** | **CCCTTACACCCACCTCCCA** | **965** | **54** | **35** |
| **Anel11Iv antisense** |  |  | **ACCTCCTTGTTAGTGGGTGC** |  |  |  |
| **Anel12Iv sense** | **GpAV20868** | **Inverse PCR** | **TACTGCATCCAGAAGGGGGA** | **1160** | **54** | **35** |
| **Anel12Iv antisense** |  |  | **ACGTCTCCGGTATCTCCTCC** |  |  |  |
| **Anel13Iv sense** | **GpAV20954** | **Inverse PCR** | **GAACCCCAAAAACGGATGCC** | **998** | **54** | **35** |
| **Anel13Iv antisense** |  |  | **GAACCCCAAAAACGGATGCC** |  |  |  |
| **Anel14Iv sense** | **GpAV20985** | **Inverse PCR** | **TCTACAGCAGCGGAGAAAGC** | **705** | **54** | **35** |
| **Anel14Iv antisense** |  |  | **GCCAATCACCACAGTTGCAG** |  |  |  |
| **Anel15Iv sense** | **GpAV21031** | **Inverse PCR** | **AAAAAGAAAGCTGGTGGAGGA** | **843** | **54** | **35** |
| **Anel15Iv antisense** |  |  | **GCCTTCGCCTGTATCTCCTC** |  |  |  |
| **Anel16Iv sense** | **GpAV21066** | **Inverse PCR** | **GACCCTGCAACACGGAAAAC** | **1124** | **54** | **35** |
| **Anel16Iv antisense** |  |  | **TCTCCTCCAGAACCTCCGAG** |  |  |  |
| **Anel17Iv sense** | **GpAV21094** | **Inverse PCR** | **TCCAAGACTGGCCTATCCGA** | **1108** | **54** | **35** |
| **Anel17Iv antisense** |  |  | **TCTCCTCCAGGCTCTCTTCC** |  |  |  |
| **Anel18Iv sense** | **GpAV21170** | **Inverse PCR** | **AAGGAAAGAGCAGCCCCTTC** | **1223** | **54** | **35** |
| **Anel18Iv antisense** |  |  | **GCCAATCACCACAGTTGCAG** |  |  |  |
| **Anel19Iv sense** | **GpAV21207** | **Inverse PCR** | **G*C*AGAACGAGAACAGCAGCTCC** | **1229** | **54** | **35** |
| **Anel19Iv antisense** |  |  | **G*C*CGGTTTCTTCGAAGCCCAGA** | |  |  |

***: phosphorothioation**
