# Supplementary material for: Virome comparisons in wild-diseased and healthy captive giant pandas
Source: Microbiome. 2017 Aug 7;5:90. doi: 10.1186/s40168-017-0308-0 (PMC5545856; doi:10.1186/s40168-017-0308-0)
Supplement: Supplementary file 2 — Genome size, GC content, and classic elements of papillomavirus identified in giant pandas. Classic elements included positions of Zinc-binding domains of E6 and E7 ORFs, LxCxE motif of E7 ORF, ATP binding site of the ATP-dependent helicase (GPPDTGKS), E2-binding site motifs, TATA box, and polyadenylation site of URR. (DOCX 18 kb) [file 40168_2017_308_MOESM2_ESM.docx]

Table S2. Genome size, GC content, and Classic elements of papillomavirus identified in giant pandas. Classic elements included positions of Zinc-binding domains of E6 and E7 ORFs, LxCxE motif of E7 ORF, ATP binding site of the ATP-dependent helicase (GPPDTGKS), E2-binding site motifs, TATA box and polyadenylation site of URR.

|  | AmPV1 | AmPV2 | AmPV3 | AmPV4 |
| --- | --- | --- | --- | --- |
| Genome size (bp) | 7,676 | 7,582 | 7,886 | 7,996 |
| GC content (%) | 43.5 | 45.6 | 58.6 | 38.5 |
| Host information | Wild/Sick | Wild/Sick | Captive/Normal | Captive/Normal |
| zinc-binding  domains (E6) | 73-183/292-402 | 76-186/295-405 | 100-210/319-429 | 73-183/292-399 |
| zinc-binding domains (E7) | none | none | 581-691 | 558-665 |
| LxCxE motif (E7) | none | none | 512-526 | 480-494 |
| ATP-dependent helicase (E1) | 1904-1927 (GPPNTGKS) | 1912-1935 (GPPNTGKS) | 2106-2129 (GPPNTGKS) | 1957-1980 (GPPNTGKS) |
| E2 binding sites (ACC(X)6/7GGT) (URR) | 7303-7314  /7339-7351  /7387-7398  /7549-7560  /7627-7638 (5) | 7247-7258  /7313-7324  /7456-7467  /7531-7542 (4) | 7383-7394/7413-7424/7544-7555/7617-7628/7651-7662/7703-7714/7758-7769/7838-7849 (8) | 7642-7653  /7814-7825  /7844-7855  /7932-7943 (4) |
| TATAbox (TATAAA) (URR) | None | None | None | 7962-7968 |
| polyadenylation site (URR) | 7168-7173  (AATAAA) | 7169-7174  (ATTAAA) | 7332-7337  (AATAAA) | 7541-7546  (AATAAA) |
