# Supplementary figures and images for: Virome comparisons in wild-diseased and healthy captive giant pandas
Source: Microbiome. 2017 Aug 7;5:90. doi: 10.1186/s40168-017-0308-0 (PMC5545856; doi:10.1186/s40168-017-0308-0)

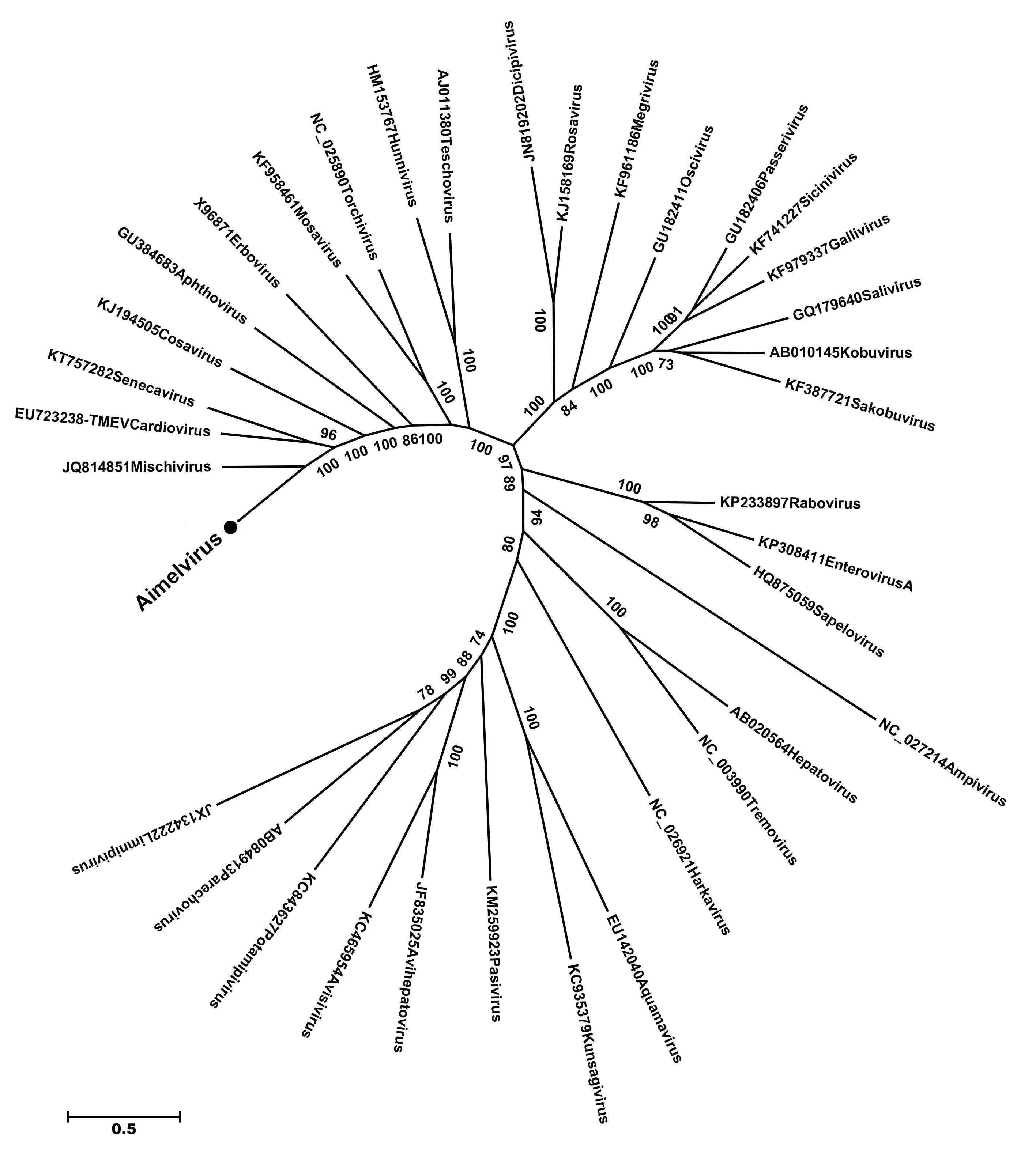

Supplement: Supplementary file 3 — Phylogenetic tree based on the P3 protein of Aimelvirus. Phylogenetic analysis was performed based on the complete amino acid sequence of P3 proteins of Aimeilvirus 1–6 and 35 representative strains of all the 35 genera in Picornaviridae. The Aimelvirus identified in this study was labeled with a black dot. (JPEG 368 kb) [file 40168_2017_308_MOESM3_ESM.jpg]

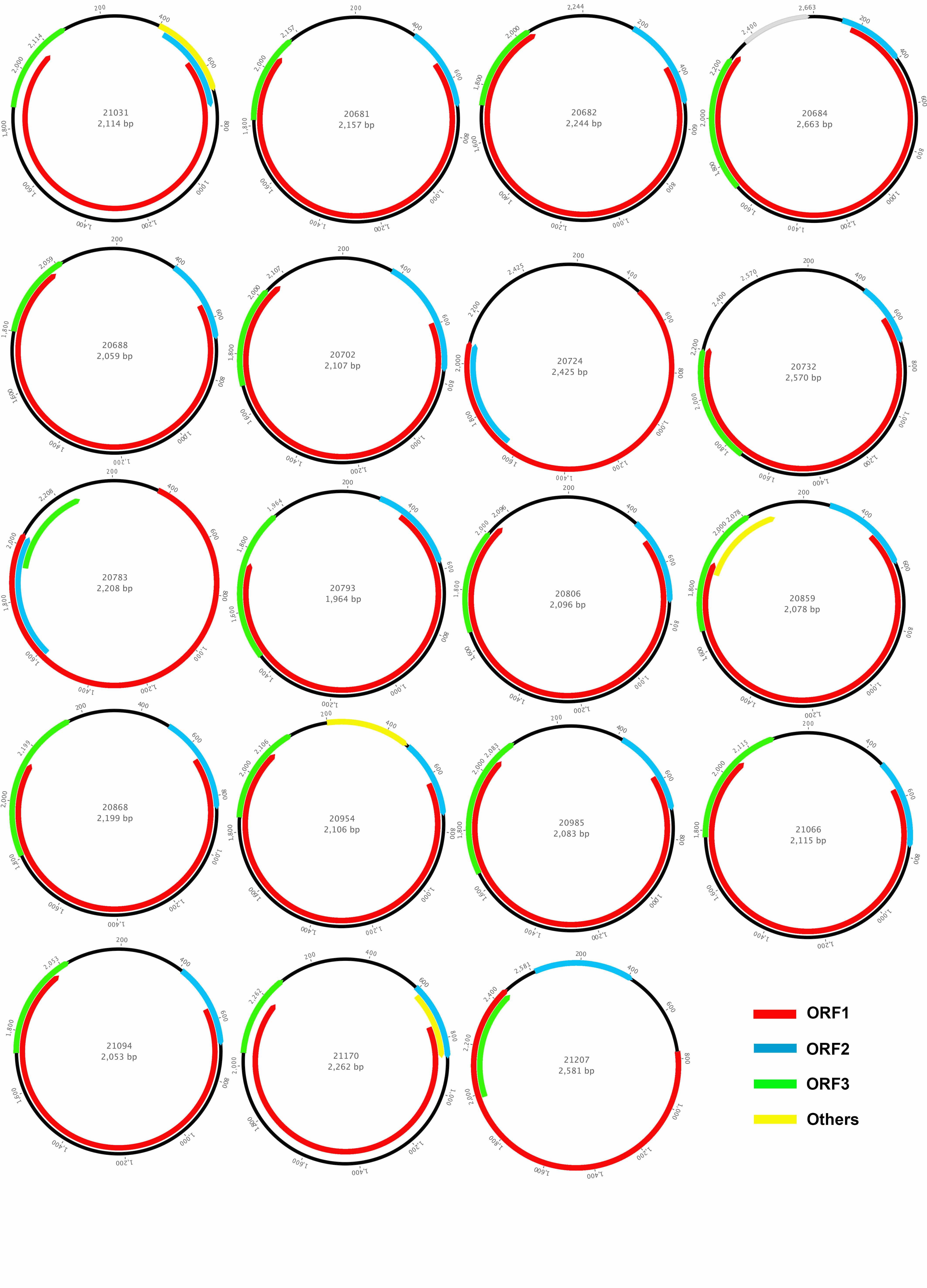

Supplement: Supplementary file 4 — Genome structure of anelloviruses identified in the present study. (JPEG 1012 kb) [file 40168_2017_308_MOESM4_ESM.jpg]
